# Nanocellulose Stabilized Pickering Emulsion Templating for Thermosetting AESO Nanocomposite Foams

**DOI:** 10.3390/polym10101111

**Published:** 2018-10-08

**Authors:** Peng Lu, Mengya Guo, Yang Yang, Min Wu

**Affiliations:** 1 Institute of Light Industry and Food Engineering, Guangxi Key Laboratory of Clean Pulp and Papermaking and Pollution Control, Guangxi University, Nanning 530004, China; lupeng@gxu.edu.cn (P.L.); guomengya@st.gxu.edu.cn (M.G.); yangyang@st.gxu.edu.cn (Y.Y.); 2 State Key Laboratory of Pulp and Paper Engineering, South China University of Technology, Guangzhou 510640, China; 3 Key Laboratory of Pulp and Paper Science & Technology of Ministry of Education/Shandong Province, Qilu University of Technology, Jinan 250353, China

**Keywords:** cellulose nanocrystals, AESO, Pickering emulsion, nanocomposite foam

## Abstract

Emulsion templating has emerged as an effective approach to prepare polymer-based foams. This study reports a thermosetting nanocomposite foam prepared by nanocellulose stabilized Pickering emulsion templating. The Pickering emulsion used as templates for the polymeric foams production was obtained by mechanically mixing cellulose nanocrystals (CNCs) water suspensions with the selected oil mixtures comprised of acrylated epoxidized soybean oil (AESO), 3-aminopropyltriethoxysilane (APTS), and benzoyl peroxide (BPO). The effects of the oil to water weight ratio (1:1 to 1:3) and the concentration of CNCs (1.0–3.0 wt %) on the stability of the emulsion were studied. Emulsions were characterized according to the emulsion stability index, droplet size, and droplet distribution. The emulsion prepared under the condition of oil to water ratio 1:1 and concentration of CNCs at 2.0 wt % showed good stability during the two-week storage period. Nanocomposite foams were formed by heating the Pickering emulsion at 90 °C for 60 min. Scanning electron microscopy (SEM) images show that the foam has a microporous structure with a non-uniform cell size that varied from 0.3 to 380 μm. The CNCs stabilized Pickering emulsion provides a versatile approach to prepare innovative functional bio-based materials.

## 1. Introduction

A nanocomposite is a multiphase material consisting of two or more components, including a matrix (continuous phase) and a discontinuous phase with at least one dimension on the nanoscale. Nanocomposites generally exhibit superior properties over their constituent materials and have a high potential for applications in various fields, including electronics, packaging, transportation, and pharmaceutical applications [1]. In particular, nanocomposite foams with porous structures are of growing interest because of their distinct characteristics such as lightweight, high strength, and multifunctional properties [2]. By far, nanocomposite foams are commonly made of polymer matrices (i.e., polyurethane, polystyrene, polyolefin, or biopolymers) reinforced with nanomaterials (i.e., montmorillonite, nanocellulose, graphenes, or carbon nanotubes) accompanying a foaming process [2,3,4,5,6,7]. However, the direct utilization of blowing agents in the foaming process is not suitable for the preparation of thin film products, and the foam morphology is hard to control, although it is suitable for large-scale productions.

In the last two decades, emulsion templating has emerged as an effective way to prepare polymer-based foams with precise morphologies [8]. In the emulsion templating method, microporous structures (pore size range: 1–100 μm) are fabricated by polymerizing the continuous phase of a concentrated emulsion followed by removal of the internal phase. Generally, the emulsion is described as one immiscible liquid dispersed in another in the form of droplets stabilized by surfactants. However, pioneering studies by Ramsden and Pickering showed that nanoparticles could irreversibly adsorb at liquid-liquid interfaces, leading to an emulsion stabilized by solid particles instead of surfactants, and this is called the Pickering emulsion system [9]. In this case, bio-based polymer foams can be produced by an appropriate selection of the stabilizing particles (i.e., nanoparticles derived from cellulose or starch) [10,11] and of the continuous phase (i.e., polylactic acid or polycaprolactone) [12,13].

Cellulose nanocrystals (CNCs) is a novel nanomaterial with a high aspect ratio (4–20 nm wide, 500–2000 nm in length) generally prepared from natural cellulose fiber. CNCs are proved to be a more sustainable and greener alternative to the petroleum-derived surfactants because they can effectively stabilize oil–water interfaces as part of a Pickering emulsion system [14,15]. CNCs have been reported to stabilize water-in-oil (*w*/*o*) emulsions [11,16,17,18] or oil-in-water (*o*/*w*) emulsions [19,20,21] for a potentially wide range of applications [22,23,24]. However, among those CNCs stabilized Pickering emulsions, research interests mainly focused on revealing the stability of *o*/*w* Pickering emulsion and behaviors of CNCs on the water-oil interface, while only a few studies reported the preparation of *w*/*o* Pickering emulsion and its application in the production of cellulose-polymer nanocomposite foams [11,16]. To get a CNCs stabilized *w*/*o* Pickering emulsion, hydrophobic modification of CNCs were generally carried out before emulsification. For instance, stable *w*/*o* Pickering emulsions were reported by tailoring surface hydrophobicity of CNCs through acetylation [25], quaternary ammonium surfactants adsorption [26,27], or organic acid grafting [17]. After that, porous cellulosic-polymer foams were prepared by polymerizing the oil phase based on Pickering emulsion template.

Nevertheless, approaches that are currently reported in the production of cellulosic-polymer foams from CNCs stabilized Pickering emulsion are complicated. For example, Blaker et al. produced renewable foams from modified bacterial cellulose stabilized Pickering emulsions, but the emulsion preparation involved a time-consuming organic phase exchange process. Moreover, the polymerization of the continuous phase of emulsions, either by thermo-polymerization reactions or photo-polymerization reactions, took more than 10 hours [16]. Another challenge that needs to be addressed in the development of Pickering emulsion-based foam materials is how to maintain the stability of the Pickering emulsion, especially in a multicomponent solvent system [28]. Therefore, exploration works in the preparation of new emulsion templates and corresponding foams are still in need.

Furthermore, as concerns around environmental issues are continually rising, increasing considerations have been taken to replace petrochemical polymer matrices with materials derived from natural resources. Acrylated epoxidized soybean oil (AESO) is a commercial derivate of soybean oil. AESO consists predominantly of triglyceride oil and can be polymerized to high molecular weights and high cross-linking thermosetting polymers [29]. AESO-based polymers have been shown to be comparable to petroleum based unsaturated polyester resins and have been used as the resin to form “green” composites or foam materials [30,31,32]. Therefore, the development of environmentally friendly, renewable, highly porous nanocomposite foams based on AESO is an attractive research topic.

In the present study, novel nanocomposite foams were obtained by polymerization of AESO continuous phases in a Pickering emulsion system stabilized by cellulose nanocrystals. Unlike reported studies that use hydrophobic modified CNCs for the preparation of Pickering emulsion, this study used original CNCs as a stabilizer without modification before emulsification. The continuous phase was a multicomponent mixture comprising AESO, 3-aminopropyltriethoxysilane, and initiator. Besides, thermosetting reactions of the Pickering emulsion were mild and fast in this research. The effects of the oil to water ratio and CNCs concentration on stability and the average droplet diameter of the Pickering emulsion were investigated. Furthermore, the structural and morphological features of the ensuing thermosetting foams were extensively studied.

## 2. Materials and Methods

### 2.1. Chemicals and Materials

CNCs were produced in our laboratory from microcrystalline cellulose (MCC) (~50 μm particle size, Avicel^®^ PH-101, Asahi Chemical Industry Co., Ltd., Tokyo, Japan). Epoxidized soybean oil (ESO) (4.9 epoxy groups per molecule), 4-Methoxyphenol (MEHQ), Triphenylphosphine (TPP), 3-aminopropyltriethoxysilane (APTS), benzoyl peroxide (BPO), and anhydrous acetone were purchased from Aladdin^®^ (Shanghai, China). Other chemical reagents used in this research were of chemical grade without any purification. Water was purified with the Milli-Q reagent system (Millipore, Burlington, MA, USA).

### 2.2. Preparation and Characterization of CNCs

CNCs ranging from 100 to 400 nm in length were obtained from high pressure homogenization of MCC. Initially, MCC water dispersion (0.5% *w*/*w*) was prepared and soaked overnight. Then, the MCC dispersion was processed using a microfluidizer processor (M-110EH30, MFIC, Westwood, MA, USA) at 30,000 PSI for 20 passes. After processing, the zeta potential of prepared CNCs was determined by a Malvern Mastersizer (ZS90X, Malvern Instruments Limited, Worcestershire, UK) from three replicate measurements to be −15.5 mv.

The morphology of CNCs was characterized by transmission electron microscopy (TEM). A drop of CNCs water suspension (0.008% *w*/*v*) was deposited on a carbon-coated electron microscope grid and then stained by 20 μL phosphotungstic acid water solution (1.5% *w*/*w*), followed by drying at 25 °C before observation. The grid was observed under the standard conditions using a TEM (HT7700, Hitachi High-Tech, Tokyo, Japan) operating at 100 kV.

Atomic force microscopy (AFM) was also used to characterize the CNCs at a 1 μm × 1 μm scan size. Innovative scanning probe microscopy (SPM) with an AFM 5000II controller (Hitachi High-Tech, Tokyo, Japan) in a tapping mode was used with a silicon probe (SI-DF40P2, f = 28 kHz, Hitachi High-Tech, Tokyo, Japan).

### 2.3. Synthesis and Characterization of AESO

AESO was synthesized in the laboratory using the following procedure. Briefly, a mixture of ESO (60 g) and MEHQ (0.14 g) was placed into a 250-mL round-bottom flask equipped with a magnetic stirrer and a reflux condenser. The reaction mixture was brought to reflux under nitrogen. Then, 10 g AA containing 0.7 g TPP was dropwise added to the mixture. The reaction mixture was kept at 100 °C for 8 h. The consumption of acrylic acid during acrylation was proved by determining the acid number and, finally, unreacted acrylic acid was removed by solvent extraction.

The analysis of transmittance FT-IR spectroscopy was performed using an FT-IR spectrometer (TENSOR II, Bruker, Ettlingen, Germany), equipped with KBr cell windows for liquid film preparations. A drop of AESO was placed between two KBr windows, then spread evenly on their surface. Subsequently, AESO absorbance spectra were obtained in the range of 400–4000 cm^−1^.

### 2.4. Emulsion Preparation and Characterization

Pickering emulsions were prepared by mixing the oil phase and water phase in different relative proportions. Typically, a mixture of AESO (10 g), APTS (1 g), BPO (0.05 g), and anhydrous acetone (0.15 g) was used as the oil phase. The water phase was a colloidal water suspension containing CNCs of 1.0–3.0 wt % with respect to the water amount. The oil phase and the water phase were promptly homogenized at room-temperature by a high shear mixer (FM200A, Fluko, Shanghai, China) at 10,000 rpm for 5 min.

The stability of the resulting emulsions was assessed by investigating the emulsion stability index. The emulsion stability index was calculated by taking the ratio between the volume of the emulsion at the time of assessment (two weeks) and the total volume of the mixture.

Optical microscopy images of the emulsion droplets were captured with a digital camera mounted microscope (Eclipse E200, Nikon, Tokyo, Japan). The images were processed using ImageJ 1.33 software. The droplet sizes and distributions were determined by measuring the diameters of four hundred droplets on several micrographs.

### 2.5. Synthesis and Characterization of AESO-Based Nanocomposite Foam

The prepared Pickering emulsion was transferred to a Teflon tube (diameter = 40 mm). Radical thermo-polymerization reactions were carried out by heating the stable emulsions at 90 °C for 60 min. The as-prepared foam was dried under vacuum at 50 °C overnight and characterized using an FT-IR spectrometer (TENSORII, Bruker, Ettlingen, Germany).

The morphology of the as-prepared foam was assessed by scanning electron microscopy (SEM) on PhenomPro (Phenom-World, Eindhoven, NL) at 10 kV.

### 2.6. Statistical Analysis

SPSS 22.0 (SPSS Statistical Software, Inc., Chicago, IL, USA) was used for conducting statistical analyses of the data to determine any significant difference between specific means at a 95% confidence interval.

## 3. Results and Discussion

### 3.1. The CNCs Material

CNC particles are regarded as an effective stabilizer in Pickering emulsion, and have been applied in various surfactant-free emulsion systems [19,20,21]. CNC particles in Pickering emulsions show a balanced interfacial wettability and are believed to irreversibly adsorb at the oil-water interface. It is reported that the aspect ratio of cellulosic nanorods directly influences their organization at the water-oil interface. The particle size of CNCs must be very small compared with the emulsion droplet size, and the shape anisotropy of CNCs allows a certain degree of flexibility when aligned along the droplet. Besides, CNCs particles have relatively high surface charge density and are prone to form well-dispersed water suspensions because of the electrostatic repulsions. Therefore, factors such as surface charge, wettability, and morphologies of CNCs could affect their ability to stabilize Pickering emulsions [33]. As this study was expected to contribute to a better understanding of the stabilization efficiency of CNCs for AESO Pickering emulsion formation, CNCs with simple morphological parameters were easily prepared from MCC through homogenization instead of other chemical approaches [15].

**Figure 1:**
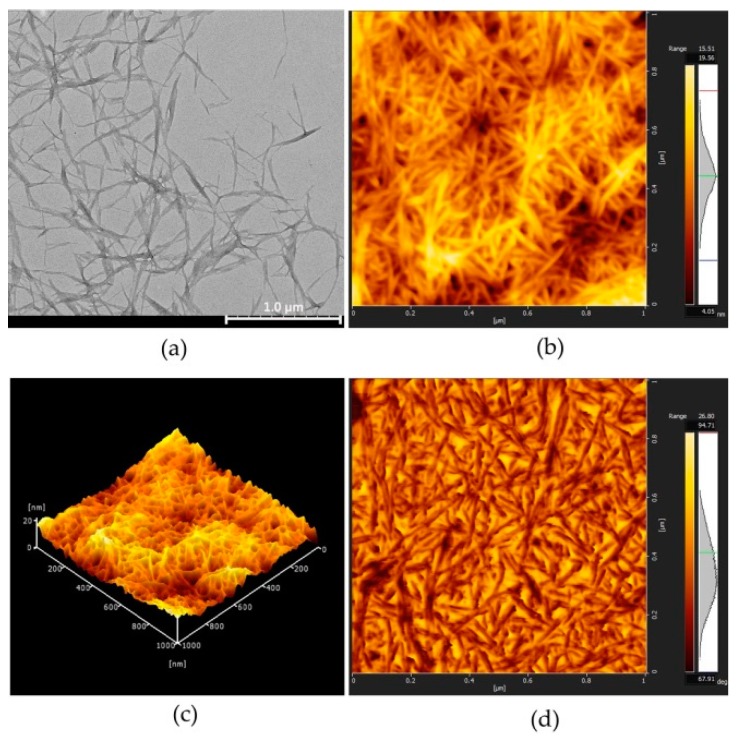
(**a**) Transmission electron microscopy (TEM) image; (**b**) atomic force microscopy (AFM) height image; (**c**) AFM 3D height image; (**d**) AFM phase image of cellulose nanocrystals (CNCs).

The morphology of the CNC particles is shown in Figure 1. Apparently, through a homogenization process, MCC is disintegrated, giving a material at least one dimension in nanoscale. TEM image showed that the CNC particles were fairly homogeneous (Figure 1a) with average sizes varying from 100 to 400 nm in length, and less than 20 nm in width. The aspect ratio of the CNCs varies from 10 to 40 with a thickness of 9.6 nm determined from AFM height image. In addition to their shapes, the zeta potential of prepared CNCs was −15.5 mv, indicating a negative charge of CNCs particles. The particle size of as prepared CNCs is in the same range as those reported in the literature [14,15], and the packing of the particles at the interface makes CNC an efficient emulsion stabilizer.

### 3.2. The Pickering Emulsion

The AESO nanocomposite foam in this study is proposed to consist of air cells discontinuously dispersed in an AESO-based polymer matrix. Based on the concept of Pickering emulsion templating, CNCs are irreversibly adsorbed at the oil-water interface and initially form stable *w*/*o* emulsions, followed by an in situ polymerization of the oil phase into the polymer matrix.

**Figure 2:**
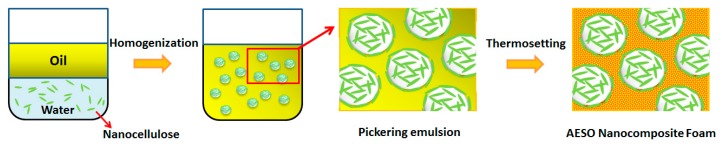
Schematic illustration of thermosetting acrylated epoxidized soybean oil (AESO) nanocomposite foam prepared from Pickering emulsion templating.

As shown in Figure 2, CNCs are initially dispersed in water and served as a water phase. For the oil phase, a mixture of 10 g AESO, 1.0 g APTS, 0.05 g BPO, and 0.15 g anhydrous acetone was used for convenience throughout the study. Under shearing forces of homogenization, the CNC nanoparticles could be evenly distributed at the oil-water interface and bent to follow the curvation of each droplet, giving rise to water-in-oil Pickering emulsions. Then, the stable Pickering emulsions are transferred to thermosetting AESO nanocomposite foams through polymerization and crosslinking reactions triggered by BPO.

The effectiveness of CNCs as stabilizers depends on factors such as particle size, inter-particle interactions, and the wettability of the particles. As hydrophilic CNCs tend to form oil-in-water emulsions [34], in order to produce water-in-oil emulsions, CNCs ought to be sufficiently hydrophobized to bend the interface toward the water phase. However, it is interesting to find that a water-in-oil emulsion was successfully prepared in this work through the use of neat CNCs without modification. The formation of *w*/*o* emulsion is likely attributed to the presence of APTS in the oil phase. During the homogenization process, CNCs particles were in contact with ATPS of the oil phase, and thus the hydroxyl groups on the CNCs might form covalent bonds with triethoxysilane groups in APTS through condensation, resulting in hydrophobic sites on CNCs surface [29]. This partial change of CNCs wettability may finally lead to the formation of a water-in-oil emulsion. Thereafter, as AESO monomers are only present in the external phase, a foam structure with a continuous polymer phase surrounding the dispersed droplets of the internal phase can be produced through polymerization. The conductivity of the CNCs stabilized AESO Pickering emulsion was determined to be 0.07 μs/cm, confirming a formation of *w*/*o* emulsion. Further confirmation of *w*/*o* emulsion can also be inferred from the SEM images of the emulsion after polymerization.

### 3.3. Effect of Oil to Water Ratio on the Emulsion Stability

**Figure 3:**
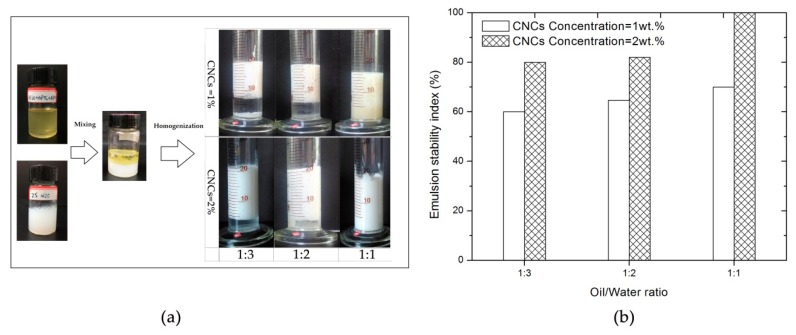
(**a**) Visual appearance of AESO Pickering emulsion prepared at different oil to water ratio; (**b**) effect of oil to water ratio on the emulsion stability index of the Pickering emulsions.

The emulsion stability is essential to the properties of the ensuing foams, and thus common influencing factors such as the oil to water ratio and concentration of CNCs were investigated to get a high stable Pickering emulsion before the polymerization reactions. The visual appearance of AESO Pickering emulsion prepared at different oil to water ratios is shown in Figure 3a. The emulsion stability is observed to be higher for emulsions stabilized by CNCs concentration of 2 wt % than that of 1 wt %. Numerous reports show that the emulsion stability increased with the increasing amount of stabilizing particles [10,18,19,26]. Increasing the amount of CNCs in the system at a constant weight fraction of oil content is believed to provide efficient coverage of the oil–water interface by CNCs and, therefore, results in good emulsion stability.

The emulsion stability index of CNCs stabilized Pickering emulsions with different oil to water ratios is presented in Figure 3b. Low emulsion stability index values were observed when the oil to water ratio was 1:3, irrespective of CNCs concentration in the water phase. The change in emulsion stability index with oil to water ratio was believed significant (*p* < 0.05) because the calculated significance level of the test was 0.01 and 0.0001 for emulsions stabilized by concentration of CNCs of 1 and 2 wt %, respectively. Earlier studies have already shown that stability of the Pickering emulsions may be influenced by the oil fraction and oil polarity [35]. In this study, we proposed that CNCs were partially modified by APTS of oil in situ during emulsification, and then adsorbed at the oil-water interface for water droplet suspension. When the oil to water ratio was 1:3, the weight fraction of water phase content is larger than that of the oil phase, and in this case, water cannot be completely dispersed in the oil phase. Consequently, a certain amount of the excess water phase will be separated from the emulsion system, resulting in a low emulsion stability index value.

In contrast, as the oil ratio increases from 1:3 to 1:1, the amount of oil phase is sufficient to wrap the water droplet to form a stable w/o emulsion, and no separated water was observed (Figure 3a). A similar trend was observed for emulsions stabilized with 2 wt % CNCs in the water phase. The emulsion stability index increased with the increasing oil to water ratio from 1:3 to 1:1. It was found that the emulsion stability index increased rapidly to 100% when the oil to water ratio reached 1:1, and moreover, the emulsion was stable to coalescence for periods over two weeks.

**Figure 4:**
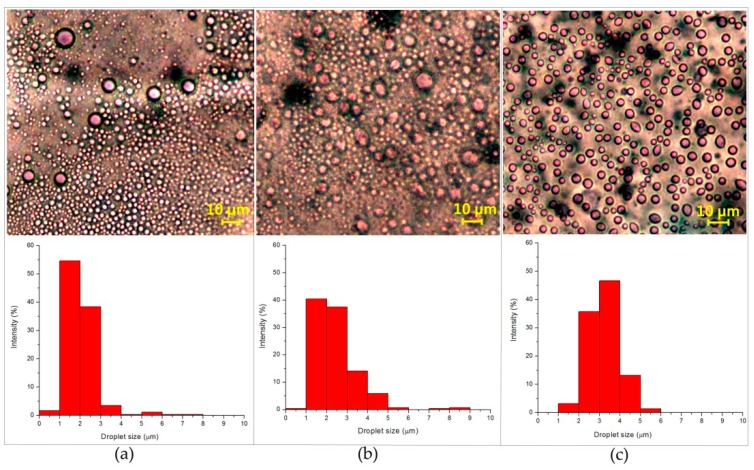
Optical micrographs (top) and droplet size distributions (bottom) of emulsions prepared at different oil to water ratios: (**a**) 1:3, (**b**) 1:2, and (**c**) 1:1. The concentration of CNCs was 2 wt % for all emulsions.

As AESO oil is less dense than water, a creaming process was systematically observed. Optical microscopy images in Figure 4 showed the trapping of the water droplet in the organic phases. Obviously, an increase in the oil to water ratio from 1:3 to 1:1 at constant CNCs concentration (2 wt %) resulted in an increase in the average drop size, but a more narrow droplet size distribution. When the oil to water ratio changed from 1:3 to 1:2, visual observation of micrographs indicates a minor shift towards larger droplets (Figure 4b). Compared with the emulsion prepared at the oil to water ratio of 1:3, the droplet in the emulsion of an oil to water ratio of 1:2 was less distinguishable in shape. However, a more stable emulsion was gained by increasing the fraction of oil from 1:2 to 1:1, together with a narrow droplet size distribution, as shown in Figure 4c. Uniformly distributed drops were identified from the optical microscopy images at an oil to water ratio of 1:1.

A previous study by Blaker showed the effect of varying water ratio on the stability and behavior of water-in-acrylated epoxidized soybean oil emulsions [16]. It was reported that the *w*/*o* emulsions underwent catastrophic phase inversion to *o*/*w* emulsions when the water ratio was increased beyond 60 vol %. This inversion may be ascribed to the formation of a water-in-oil-in-water multiple emulsion, in which the dispersed-phase fraction is increased by the continuous inclusion of water droplets in the oil until a critical value is reached and the inversion is triggered [36,37]. Nevertheless, as the oil phase in this study is a multiphase system consisting AESO/APTS/CNCs/water, the reasons for the triggering of the change of emulsion stability and droplet size distribution are still not clear and remain to be understood.

### 3.4. Effect of Concentration of CNCs on the Emulsion Stability

**Figure 5:**
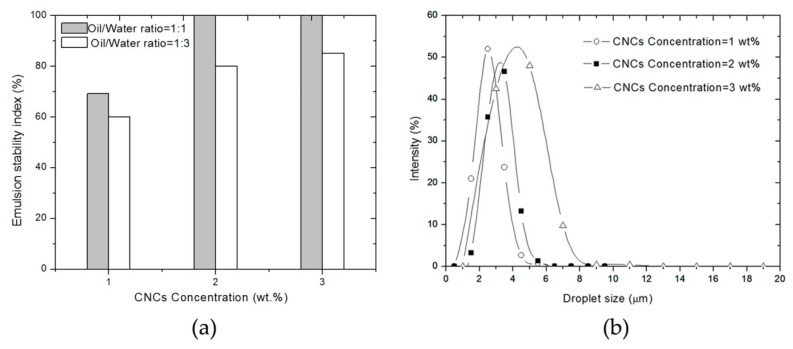
(**a**) Effect of concentration of CNCs on emulsion stability index; (**b**) droplet distributions of emulsions prepared at different CNCs concentration with a fixed oil to water ratio at 1:1.

Several publications indicate that the stability of particle-stabilized emulsions depends on the particle concentration and particle-particle interaction [19,38]. Figure 5a shows the change of the emulsions stability index with the concentration of the CNCs in the water phase. In all cases, regardless of the oil to water ratio, the emulsion stability index for the emulsions increased with increasing concentration of CNCs.

At CNCs concentrations below 2 wt %, the oil and water phases separate and the emulsion stability index was low. In order to get a stable emulsion, CNCs should be irreversibly adsorbed on the oil-water interface, and a minimum coverage ratio is required to efficiently stabilize a drop [38]. As a result, at low CNCs concentration, only a part of the water volume will be emulsified, especially at a low oil to water ratio (1:3). However, the emulsion stability index increased with the added CNCs, and complete emulsification was reached at CNCs concentration of 2 and 3 wt %, respectively.

In Figure 5b, dispersed droplets with a small size and narrow distribution were obtained at low concentrations of CNCs. Droplet sizes increased with increasing concentration of CNCs at a constant oil to water ratio, with a corresponding increase in stability against sedimentation. For the samples prepared with high CNCs concentrations, at and above 2 wt %, a stable emulsion of droplet diameter around 3–5 μm was obtained. Large droplet size and wide droplet size distribution generally show a tendency to coalescence, as shown in previous reports from other types of Pickering emulsions [18,39,40]. Nevertheless, at high particle concentrations, the particles are adsorbed irreversibly at the interface and will act as a mechanical barrier against droplet coalescence. In addition to this, the interconnection of CNCs between droplets may form a three-dimensional network to impede the coalescence of the dispersed droplets [37]. When the concentration of CNCs increased to 3 wt %, the CNCs were in excess apart from adsorbing at the oil-water interface. The remaining CNCs dispersed in the aqueous phase involved in entanglements and interconnecting droplets, promoting an interconnected network of droplets and, consequently, higher emulsion stability, irrespective of the relatively large droplet size in this case.

It is concluded that the stability of the Pickering emulsion is controlled by the amount of CNCs introduced and the oil to water ratio. In this study, 2 wt % of CNCs, together with an oil to water ratio of 1:1, were sufficient to form stable emulsions and individual droplets could be obtained.

### 3.5. The AESO-based Nanocomposite Foam

**Figure 6:**
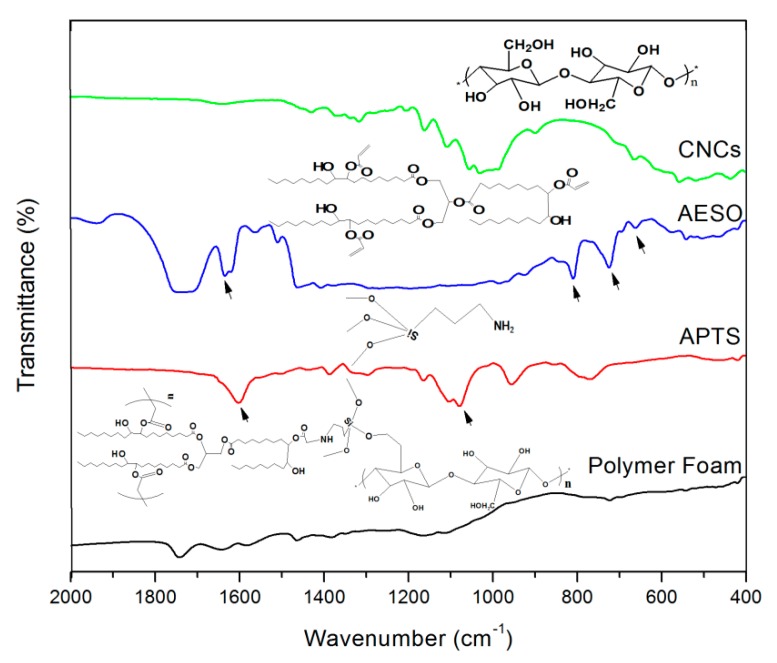
FT-IR spectra of CNCs, AESO, 3-aminopropyltriethoxysilane (APTS), and nanocomposite polymer foam.

AESO has a sufficient number of double bonds to go through chain propagation and form a polymer network by radical polymerization [29]. In addition, the double bonds in the AESO structure could react with the amino ends in the APTS through a Michael addition reaction [41], and thereafter crosslink with adjacent AESO or APTS to form a network structure. The success of the foams preparation was demonstrated by FT-IR, as shown in Figure 6.

In AESO spectra, the characteristic peak at 610–690 cm^−1^ was assigned to C–H wagging vibration of vinyl hydrocarbon compounds R–CH=CH_2_, the =CH_2_ twisting vibration at 800–810 cm^−1^, and C=C stretching vibration at 1620–1640 cm^−1^ was assigned to H_2_C=CH– (C=O)–O–R in AESO structure. For APTS, the primary amine was proven by the N–H deformation vibration peaks at 1580–1650 cm^−1^, and broad N–H out-of-plane bending vibrations at 650–900 cm^−1^. A symmetrical Si–O–C stretching vibration at 945–990 cm^−1^, plus the symmetrical Si–O–C stretching vibration at 1070–1100 cm^−1^ and 1140–1190 cm^−1^, were related to (–Si–O–CH_2_CH_3_) of APTS. After the thermosetting process, the spectrum of C=C double bonds at 800–810 cm^−1^ and 1620–1640 cm^−1^ disappeared in the nanocomposite foam, implying the chain propagation of AESO. Moreover, typical peaks appeared at 1740–1770 cm^−1^ (C=O stretching vibration of R–C=O–O–R) and 1450–1480 cm^−1^ (C–H symmetrical and deformation vibration on –N–CH_2_–), reflecting the successful covalent coupling of AESO and APTS (–O(C=O)–CH_2_–CH_2_–NH–), through the Michael addition reaction.

**Figure 7:**
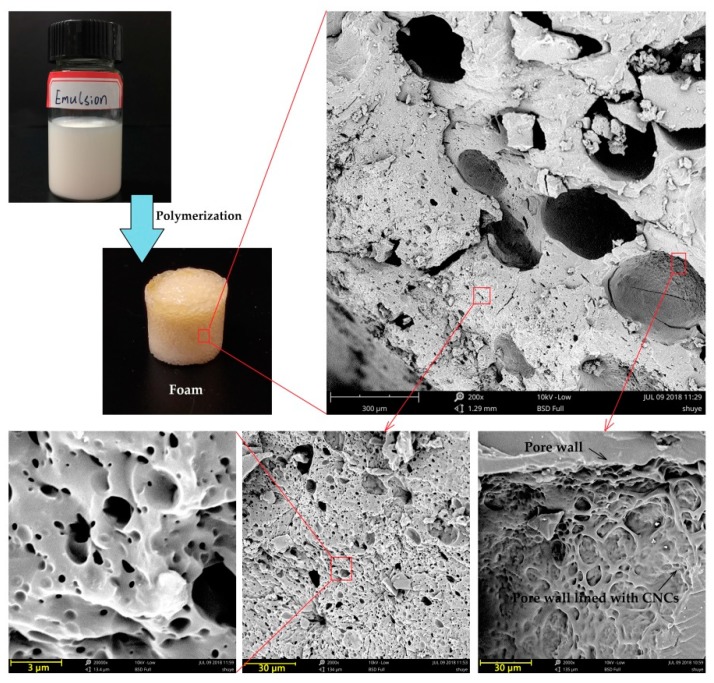
The visual appearance of AESO nanocomposite foam and corresponding scanning electron microscopy (SEM) images at different magnification. The foam is derived from a Pickering emulsion with 2 wt % of CNCs and an oil to water ratio of 1:1.

A typical porous structure in the AESO nanocomposite foam is revealed by scanning electron microscope (SEM) micrographs in Figure 7. SEM images show that the polymerization of AESO in the external phase of the Pickering emulsion resulted in a microporous structure.

The fresh prepared AESO Pickering emulsion in Figure 7 is visibly smooth and uniform without any observable coarse cream. After polymerization at 90 °C for 60 min, AESO nanocomposite foam with porous structure was obtained. Detailed pore structures of the foam from SEM demonstrate that the foam contains interconnected pores of variable size scattered among a continuous polymer matrix. There are a small number of large pores with pore size varying from 187 to 380 μm. These large pores have a closed cell structure and the CNCs can clearly be seen lining the pore walls. However, the size of the pores is significantly larger than the droplet size of the emulsion. Mechanisms of this complex phenomenon were still under investigation. A probable assumption may be the loss of thermostability and coalescence of the emulsion under the thermopolymerization process. In addition, a large number of small pores varying in the range of 0.3–2.4 μm diameter were observed. These small pores consist of both close-cell structure and open-cell structure, as indicated by the SEM image at 20,000 magnification. The formation of these small pores is hard to understand because CNCs were not observed on the cell wall. A more detailed investigation is needed to reveal the exact mechanisms that are at play.

## 4. Conclusions

In this study, an AESO thermosetting nanocomposite foam was prepared by nanocellulose stabilized Pickering emulsion templating. With CNCs as the stabilizer, water droplets were well dispersed into reactive oil phase comprising AESO, APTS, and BPO. The stability of the Pickering emulsion was influenced by the oil to water weight ratio and the concentration of CNCs. The emulsion prepared at an oil to water ratio of 1:1 and a concentration of CNCs of 2.0% showed good stability during the two-week storage period. The ensuing nanocomposite foams were formed by heating the Pickering emulsion at 90 °C for 60 min. SEM images show that the foam has a microporous structure with non-uniform cell size varying from 0.3 to 380 μm. The Pickering emulsion templating based on CNCs stabilized AESO was proven to be an effective way to prepare the microporous bio-based nanocomposite.
